# Targeted therapies for cancer during the COVID-19 pandemic: a threat or a blessing?

**DOI:** 10.2217/pgs-2020-0059

**Published:** 2020-06-10

**Authors:** Elissar Moujaess, Hampig Raphael Kourie, Joseph Kattan

**Affiliations:** ^1^Department of Hematology & Oncology, Faculty of Medicine, Saint Joseph University, Beirut, 17-5208, Lebanon

**Keywords:** cancer, COVID-19, neoplasm, SARS-CoV-2, targeted therapy

The COVID-19 pandemic has had significant consequences on the healthcare system since its outbreak in China at the end of December 2019, particularly in cancer care [1–3]. Many authors have already highlighted the dilemma that oncologists face in the management of their patients in the current situation [4–6]. In a cohort of 1590 COVID-19 patients, Liang *et al.* evaluated 18 patients with cancer. According to their data, the incidence of COVID-19 in this population was higher than that in the general population, and cancer patients who were infected had poorer outcomes [7]. The authors concluded that it is safer to postpone elective surgeries and adjuvant chemotherapy in stable patients. Two other cohort studies in China yielded results that are compatible with those of Liang *et al.* regarding the worse outcome of cancer patients who acquire the COVID-19 [8,9]. The authors of one of these studies, Zhang *et al.* proposed to postpone immunosuppressive therapies or at least, administer them with dose reduction during the pandemic [8]. More recently data from a larger cohort that assessed 641 patients of whom 105 had cancer in multiple centers of Hubei, China, confirmed that worse outcomes are observed in cancer patients, especially those who underwent recent surgery [10]. Similarly, an analysis of 334 patients with cancer, among 5668 patients with COVID-19 in New York city demonstrated a higher need for intubation in these patients but without significant difference in mortality rate compared with the general population [11].

In spite of the above observations, one should think about the risk of interrupting cancer treatment for a prolonged period of time as well. This was highlighted by the TERAVOLT registry that collected and analyzed data on thoracic cancer patients who were infected with COVID-19 and concluded that mortality rates, although high, were not correlated with any specific type of cancer treatment [12]. Thus, some papers encourage clinicians to have a more sensible approach and pursue effective cancer treatment [13].

There is no doubt that oncologists must make all efforts to protect their patients from a COVID-19 infection. However, we believe that the data reported above must be cautiously interpreted and cannot be extrapolated to all cancer patients. Recently, multiple recommendations issued by health or governmental authorities, pointed to targeted therapies as immunosuppressive category of drugs that warrant the same precautions as with chemotherapy [14]. In fact, targeted agents constitute a newer therapeutic tool that followed surgery and chemotherapy for cancer. They are substances that target specific molecules within the tumor cells or their microenvironment, and exhibit various function such as interfering with cycle progression, cell death, metastasis or angiogenesis according to their target (cell surface antigens, growth factors, signal transduction pathways) [15]. The risk of infectious complications exists but is variable with targeted therapy. According to Reinwald *et al.* kinase inhibitors that target the mTOR, Janus kinase and BCR pathways exhibit an increased risk of infectious, sometimes fatal complications; while this risk is minor with kinase inhibitors of the angiogenesis-related growth factors [16]. Another problem with targeted agents is that they can be responsible of pulmonary adverse reactions such as interstitial pneumonitis, alveolar hemorrhage and acute respiratory distress syndrome that cannot be easily differentiated from a COVID-19 infection, neither clinically nor radiographically [17].

We did not find enough evidence on the risk of COVID-19 in patients treated with targeted therapy in the literature. Conversely, some available data indicate that targeted therapy can be harnessed for the treatment of the novel coronavirus.

## Insufficient data on the impact of COVID-19 on patients treated with targeted agents

In the Liang *et al.* cohort, most of the cancer patients that were included were treated with chemotherapy or underwent a recent surgery. None of the patients were on a targeted therapy [7]. Whether chemotherapy and surgery predispose patients to a more immunocompromised state than targeted therapy is not well elucidated in the literature, but it is known that various targeted agents act differently *in vitro* on antitumor immunity [18]. As for the cohort of Zhang *et al.* [8] that included 28 cancer patients, an exposition to any cancer treatment (chemotherapy, radiotherapy, targeted therapy or immunotherapy) within 14 days prior to the infection was associated with a poor outcome. The study included two patients who were receiving a targeted therapy within this time frame. On the other hand, almost 28% of the patients were thought to have a hospital-acquired infection, whereas many targeted agents are administered via oral route and can spare hospital visits and in-hospital transmission of the virus.

Jing *et al.* assessed 12 of 1524 cancer patients who tested positive for COVID-19, of whom one patient was treated with osimertinib for non-small-cell lung cancer and had mild symptoms and no adverse outcomes [9]. Clinicians are encouraged not to withhold targeted therapy for good responders rather than pursuing it where not indicated; and this principle is also true irrespective of the pandemic. In fact, we found that COVID-19 could be cured with antiretroviral therapy in a patient with lung adenocarcinoma without the need to discontinue his therapy with osimertinib [19], and that good responders to tyrosine kinase inhibitors in chronic myelogenous leukemia are less likely to develop symptoms of SARS-CoV-2 than nonresponders [20].

## Data on the investigational use of targeted agents in the treatment of COVID-19

Cancer research was affected by the emerging of COVID-19. Many centers had to slow their activity due to public health measures [21], whereas others have shifted their activity toward investigating available drugs in the treatment of the novel coronavirus [22]. In fact, a neural network analysis of virus–host interaction retrieved available drugs that can potentially act against the SARS-CoV-2. Among these drugs more than one targeted agent widely used in cancer treatment were found, particularly a tyrosine kinase inhibitor (afatinib) and a proteasome inhibitor (ixazomib) [23]. The mTOR kinase inhibitor rapamycin was also deemed repurposable for COVID-19 treatment by another large network proximity analysis [24]. Furthermore, previous studies have highlighted the use of kinase inhibitors in the reduction of virus infectivity and this led some researchers to investigate the role of antityrosine kinase such as sunitinib and erlotinib in the treatment of COVID-19 [25]. These studies are certainly investigational on a molecular basis and clinical applicability needs additional trials. Nonetheless, the fact that targeted therapy is being evaluated in COVID-19 treatment must encourage clinicians to pursue these agents during the pandemic, especially for good responders, until further notice. We identified one ongoing randomized controlled trial that is evaluating the role of the anti-angiogenic agent bevacizumab in the treatment of critically ill patients with COVID-19 in China (NCT04275414) [26]. This study is still recruiting, and no preliminary results are reported yet, but it still emphasizes that the use of targeted agents can be promising in the therapy for COVID-19.

In the end, there is a trend during this pandemic toward withholding or postponing treatments where possible in the oncology practice based on data from small cohorts. We believe that these data are insufficient to modify the management of patients treated with targeted therapy because this population is not well represented in the studies done to date. While some targeted anticancer agents are investigated in the treatment of COVID-19, should oncologists prioritize the use of targeted therapy for cancer treatment where applicable as a protective strategy in the era of COVID-19? The answer to this question is not evident and needs studies performed on larger scales.
